# Plasma pTau181 as a biomarker for Alzheimer's disease

**DOI:** 10.1002/mco2.1

**Published:** 2020-05-22

**Authors:** Jie Meng, Peng Lei

**Affiliations:** ^1^ Department of Neurology and State Key Laboratory of Biotherapy West China Hospital Sichuan University Chengdu China

Alzheimer's disease (AD) is one of the most difficult neurological disorders, because there are no means of biomarkers to predict the disease progression, nor is there a cure. Presently, two pathological hallmarks of AD, amyloid plaques and tangles (which were formed by amyloid‐β, Aβ, and tau), can be visualized by positron emission tomography (PET), and Aβ or tau levels in cerebrospinal fluid (CSF) and plasma can be measured.^1^ However, the approved methods, with their disadvantages of high cost and relative invasiveness, are more for diagnosis rather than disease prediction. Development of a blood‐based test for AD that can be used as a biomarker as well as a diagnostic tool would be ideal and is critical for drug development.

Recently, Thijssen and colleagues^1^ have performed a clinical trial to measure plasma levels of phosphorylated tau181 (pTau181) in various tauopathies, built on early work suggesting that plasma pTau181 can differentiate AD from healthy controls.^2^ In the current study, the authors have examined plasma pTau181 in eight groups classified by their disease diagnosis, with a total of 362 individuals aged from 58 to 70. The cohort includes cases of healthy control (HC; n = 69), mild cognitive impairment (MCI; n = 47), AD (AD_clin_; n = 56), and different types of frontotemporal dementia (corticobasal syndrome [n = 39], progressive supranuclear palsy [n = 27], behavioral variant frontotemporal dementia [n = 50], nonfluent variant primary progressive aphasia [n = 27], and semantic variant primary progressive aphasia [n = 26]). The plasma level of pTau181 was 3.5‐fold elevated in AD_clin_ compared to HC, with an intermediate increase in the MCI group. In contrast, all cases of frontotemporal dementia have the same range of plasma pTau181 as HC, which indicates that plasma pTau181 differentiated patients with clinically diagnosed AD from other tauopathies and elderly controls. Therefore, pTau181 may be a specific diagnostic marker for AD.

In addition, the authors have found that higher plasma pTau181 can predict the rate of cognitive decline in AD_clin_/MCI groups with a 2‐year following‐up in AD_clin_/MCI groups. They have also measured plasma Aβ42/Aβ40, a previously suggested assay to identify brain amyloidosis in individuals with or at risk for AD,^2^ and found that the fold changes in plasma pTau181 between groups are significantly higher than Aβ42/Aβ40. The analysis also revealed that plasma Aβ42/Aβ40 ratio was a less accurate indicator of cognitive impairment than plasma pTau181 level.

In the same issue of Nature Medicine, Janelidze et al^3^ also have conducted a similar trial investigating plasma pTau181. They have studied plasma pTau181 of a total 589 individuals and divided into three cohorts, which are cohort 1 (n = 182), cohort 2 (n = 344), and cohort 3 (the neuropathology cohort included 16 autopsy‐con‐firmed AD dementia and 47 autopsy‐confirmed non‐AD individuals). Each cohort was designed to similarly include cognitively unimpaired participants, MCI patients, AD dementia, and non‐AD neurodegenerative disease patients. Additionally, cohort 1 was assigned with all tau PET imaging cases, and longitudinal follow‐up cases over 8 years were assigned into cohort 2.

They reported that plasma pTau181 is significantly elevated in people with AD compared to control individuals. Such elevation starts at the early stage of AD (Braak stage I‐II), and continues to increase throughout the progress (Braak stage V‐VI), which also correlates with CSF pTau181 elevation. In Aβ PET^+^ patients, the plasma pTau181 is weakly correlated with CSF pTau181, but strongly correlates with tau PET status as well as Aβ PET status, suggesting that this parameter is related to disease pathology.

Interestingly, plasma pTau181 was found to be increased in people who convert to AD dementia from MCI, compared with those who converted to dementia due to non‐AD diseases. These results are promising to utilize plasma pTau181 as an AD biomarker to identify patients that likely to be tau PET^+^ instead of an actual test using PET, or to distinguish AD dementia from other forms of dementia.

In summary, both studies have provided strong evidence that plasma pTau181 may serve as a possible predictive and diagnostic biomarker with reasonable sensitivity and accuracy. The fact that they both found that plasma pTau181 accurately predicts the transformation to AD from people without dementia, highlighting the possibility that plasma pTau181 may be valuable biomarkers for clinical trials.

Janelidze et al reported that the correlations with CSF pTau181 and plasma pTau181 can only be observed in Aβ PET^+^ groups. They also found that plasma pTau181 significantly varies in the Aβ PET^–^ group comparing with CSF pTau181 levels, indicating that CSF and plasma pTau181 may be differentially regulated by Aβ pathological status. In addition, the plasma pTau181 initiated to increase around the time of Aβ PET positivity, and continued to increase as Aβ accumulates. Recently, Nicolas et al quantified tau phosphorylation in 370 participants with 8‐year following‐up, and have provided clinical evidence that pTau181 co‐elevated with aggregated Aβ two decades before the development of tau pathology.^4^ Interestingly, it was previously reported in mice that Aβ accelerates tangle formation,^5^, ^6^ and loss of tau prevents Aβ toxicity.^7^, ^8^, ^9^ These observations point to a link between altered Aβ and tau metabolism, and supports the notion that both proteins may interact during the course of the disease manifestation.

In addition, the plasma pTau181 was found to correlate with CSF pTau181 in this study. However, such a correlation between plasma and CSF is missing in total tau measurement,^3^, ^10^ indicating that there could be a differentiated regulatory mechanism for plasma tau and its phosphorylation, which may need further investigation.

In conclusion, Boxer et al and Janelidze et al have collectively discovered an advanced diagnostic biomarker, plasma pTau181, for Alzheimer's disease. Such blood test is less invasive and costly compared to the current gold standard, PET imaging, and CSF sample testing (Figure 1), which may significantly facilitate our future investigations on drug development for Alzheimer's disease.

**FIGURE 1:**
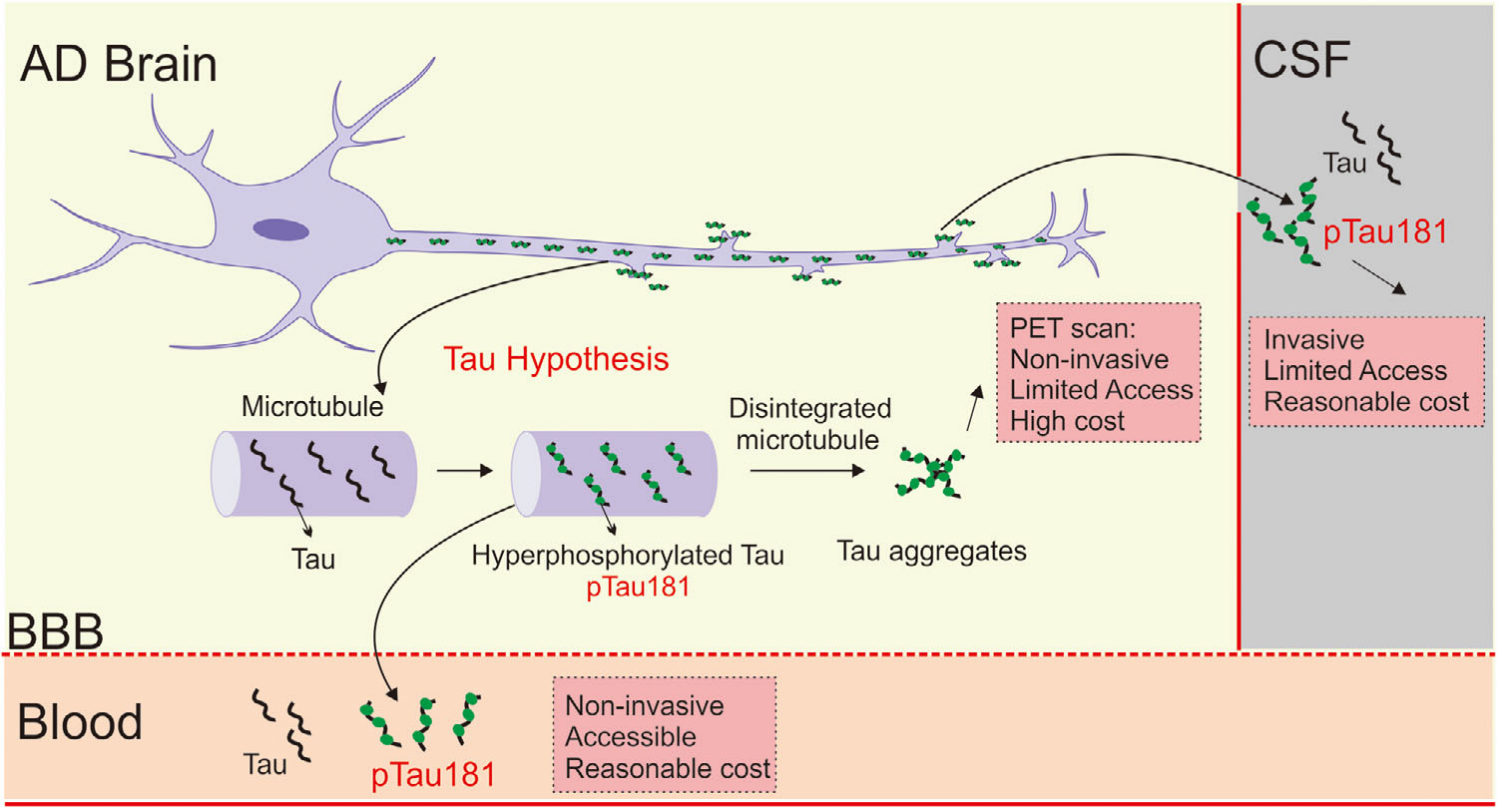
The potential of plasma tau as Alzheimer's disease (AD) biomarker. In the AD brain, hyperphosphorylated tau affects the integrity of microtubule in neurons, resulting in intracellularly tau aggregation and toxicity. With the advance of technology, we can measure tau aggregation in the brain (by PET) and tau/phosphorylated tau 181 levels in cerebrospinal fluid (CSF) and plasma. Each method has its advantages and disadvantages, but pTau181 may be a better candidate for AD biomarker.

## CONFLICT OF INTEREST

The authors declare no conflict of interest.
